# Direct interaction of FliX and FlbD is required for their regulatory activity in *Caulobacter crescentus*

**DOI:** 10.1186/1471-2180-11-89

**Published:** 2011-05-02

**Authors:** Zhaohui Xu, Rachel J Dutton, James W Gober

**Affiliations:** 1 Department of Chemistry and Biochemistry and Molecular Biology Institute, University of California, Los Angeles, CA 90095-1569, USA; 2 Department of Biological Sciences and Center for Photochemical Sciences, Bowling Green State University, Bowling Green, OH 43403-0208, USA; 3 FAS Center for Systems Biology, Harvard University, 52 Oxford Street, Cambridge, MA 02138, USA

## Abstract

**Background:**

The temporal and spatial expression of late flagellar genes in *Caulobacter crescentus *is activated by the transcription factor FlbD and its partner trans-acting factor FliX. The physical interaction of these two proteins represents an alternative mechanism for regulating the activity of σ^54 ^transcription factors. This study is to characterize the interaction of the two proteins and the consequences of the interaction on their regulatory activity.

**Results:**

FliX and FlbD form stable complexes, which can stand the interference of 2.65 M NaCl. The stability of FliX and FlbD was affected by the co-existence of each other. Five FliX mutants (R71A, L85K, Δ117-118, T130L, and L136K) were created by site-directed mutagenesis in conserved regions of the protein. All mutants were successfully expressed in both wild-type and *ΔfliX Caulobacter *strains. All but FliX_L85K _could rescue the motility and cell division defects of a *ΔfliX *mutant strain. The ability of FliX to regulate the transcription of class II and class III/IV flagellar promoters was fully diminished due to the L85K mutation. Co-immunoprecipitation experiment revealed that FliX_L85K _was unable to physically interact with FlbD.

**Conclusions:**

FliX interacts with FlbD and thereby directly regulates the activity of FlbD in response to flagellar assembly. Mutations in highly conserved regions of FliX could severely affect the recognition between FliX and FlbD and hence interrupt the normal progression of flagellar synthesis and other developmental events in *Caulobacter*.

## Background

*Caulobacter crescentus *undergoes a series of programmed differentiation events within each cell cycle and generates two dissimilar progeny cells, a motile swarmer cell possessing a single polar flagellum and a sessile stalked cell. A hallmark of this asymmetric cell division event is the temporal expression and asymmetric targeting of regulatory proteins as well as proteins comprising cellular structures such as the flagellum [1-5]. Over fifty genes are required for flagellar biogenesis in *C. crescentus*, and their temporal and spatial expression is regulated by both cell cycle events and the progression of flagellum assembly. Epistasis experiments have revealed that flagellar gene expression is subject to a regulatory hierarchy that reflects the assembly sequence of major flagellum sub-structures [6-15]. The expression of the early flagellar genes (class II) encoding components of basal body switch, MS-ring, and flagellum-specific type-three secretion system (TTSS) is regulated by the timed synthesis and phosphorylation of the transcription factor CtrA [16-18]. The polar assembly of the MS-ring/switch/TTSS complex is required, in turn, for the transcription of genes (class III) encoding structures such as the rod, outer membrane rings, and the hook [8,10,13,14]. Finally, the complete construction of these class III-encoded structures are required to derepress the translation of flagellin mRNA (class IV), leading to the assembly of flagellar filament structure [19-22]. Thus, during *C. crescentus *flagellar biogenesis two different regulatory checkpoints link structural assembly to flagellar gene expression.

The transcription of class III and IV flagellar genes requires σ^54^-containing RNA polymerase and the DNA binding protein, integration host factor (IHF) [23-28]. Transcription of these flagellar genes is under cell cycle control and, late in the cell cycle, is restricted to the swarmer cell compartment of the predivisional cell. This temporal and spatial transcription is regulated by FlbD, a σ^54 ^transcription factor [29-34]. The conserved receiver domains of this class of proteins are usually phosphorylated by a cognate sensor histidine kinase, which in turn stimulates oligomerization and DNA-binding of these proteins at enhancer sequences. Rather than phosphrylation, FlbD activity is regulated by FliX, a conserved trans-acting factor that is present in polarly flagellated α-proteobacteria and has no demonstrated histidine kinase activity [35-38]. *Caulobacter *strains bearing mutations in *fliX *are non-motile and do not transcribe class III and IV flagellar genes. Gain-of-function mutations in FlbD can by-pass the transcriptional requirement for FliX, suggesting that FliX is a trans-acting factor rather than a structural component of the flagellum [36]. Additionally, FliX enhances FlbD-activated transcription *in vitro *by stimulating purified FlbD to form higher-order oligomers [35]. Interestingly, overexpression of FliX suppresses FlbD-activated transcription *in vivo*, and a mutant allele of *fliX*, *fliX 1*, has been isolated that can by-pass the early flagellar assembly requirement for class III and IV transcription [38]. These observations suggest that upon the complete assembly of an early class II flagellar basal body structure, FliX switches from a negative to a positive regulator of FlbD.

The physical interaction of FliX and FlbD represents a novel mechanism for regulating the activity of a σ^54 ^transcription factor [35]. Here, we describe a genetic and biochemical analysis dissecting the role of FliX in regulating FlbD activities. We present evidence that FliX and FlbD are in stable complexes under physiological conditions. Furthermore, we show that highly-conserved regions of FliX are critical for its productive interaction with FlbD and for proper regulation of flagellar gene expression in response to the progression of flagellar assembly.

## Methods

### Bacterial strains and plasmids

Bacterial strains and plasmids involved in this work are summarized in Table 1. *Caulobacter crescentus *strains were grown in peptone-yeast extract (PYE) [39] at 31°C. Antibiotics were supplemented when necessary to a final concentration of 2.5 μg/ml of chloramphenicol, 2 μg/ml of tetracycline, or 20 μg/ml of nalidixic acid. PYE motility plates contained 0.3% (w/v) agar. *E. coli *strains were grown at 37°C in Luria-Bertani broth supplemented with one or more of the following antibiotics: chloramphenicol (30 μg/ml), tetracycline (12.5 μg/ml), or ampicillin (50 μg/ml). DNA manipulations were carried out according to standard procedures. Plasmids were introduced into *C. crescentus *by conjugation with *E. coli *S17-1.

**Table 1:** Bacterial strains and plasmids used in this study

Strains or plasmids	Genotypes or descriptions	Sources
***C. crescentus***		
LS107	*syn-1000*, *bla-6*, amp^s ^derivative of NA1000	Stephens *et al*. [45]
JG1172	*syn-1000 bla-6 ΔfliX*	Muir *et al*. [38]
SC1032	*flbD198*::Tn5	Ohta *et al*. [41]
		
***E. coli***		
S17-1	Rp4-2, Tc::Mu, Km::Tn7	Simon *et al*. [46]
BL21(DE3)	F^-^*ompT gal [dcm] [lon] hsdS_B _(*r_B_^- ^m_B_^-^*; *an *E. coli *B strain) with DE3, a *λ *prophage carrying the T7 RNA polymerase gene	Novagen
		
**Plasmids**		
pX21b	derivative of pET-21b carrying histidine-tagged FliX under the control of T7 promoter, Ap^r^	Muir & Gober [36]
pBBR1MCS	broad host range cloning vector, multicopy, Cm^r^	Kovach *et al*. [47]
pZXfliX	derivative of pBBR1MCS carrying PCR generated 894 bp BamHI-HindIII fragment containing *fliX *gene and its promoter	This study
pZX71A	derivative of pZXfliX, codon 71 cgcc→gcc	This study
pZX85K	derivative of pZXfliX, codon 85 ctg→aag	This study
pZXΔ117-8	derivative of pZXfliX, deletion of codon 117 and 118	This study
pZX130L	derivative of pZXfliX, codon 130 acc→ctg	This study
pZX136K	derivative of pZXfliX, codon 136 ctg→aag	This study
pfliX1	derivative of pBBR1-MCS expressing *fliX 1*, an allele of *fliX *carrying a point mutation at the sixteenth codon (R16G), a frame shift at codon 141, and an extended carboxyl terminus of 67 amino acids	Muir *et al*. [38]
pfliF/lacZ/290	*fliF-lacZ *transcriptional reporter vector, Tc^r^	Wingrove & Gober [48]
pfliK/lacZ/290	*fliK-lacZ *transcriptional reporter vector, Tc^r^	Gober & Shapiro [25]

### Identification of FliX-bound proteins with mass spectrometry

About 1.64 g of CNBr-activated sepharose 4B beads (GE Healthcare, Piscataway, NJ, USA) were swelled and washed as recommended by the manufacture and incubated overnight with 36.6 mg of histidine-tagged FliX (FliX-His) that was prepared as previously described [35]. After incubation at 4°C with end-over-end rotation, the bead complexes were alternately washed with acidic buffer (0.1 M acetate, 0.5 M NaCl, pH 4.0) and alkaline buffer (90 mM Tris·Cl, 0.5 M NaCl, pH 8.5) for 3 cycles. Such prepared sepharose-FliX complexes were then conditioned by PBS buffer (0.1 M sodium phosphate, 0.15 M NaCl, pH 7.2) and stored at 4°C for later use. Meanwhile, 5 liters of *C. crescent *LS107 culture was harvested by centrifugation, resuspended in 100 ml of PBS buffer, lysed by French Press, and centrifuged at 26,690 *g *for 1 h. The supernatant was mixed with the above sepharose-FliX complexes and incubated at 4°C for overnight with gentle rocking. Cell extract was then removed by centrifugation. The pellet containing the sepharose bead complexes was washed with 20 ml of PBS buffer for three times and resuspended in 5 ml of the same buffer. An aliquot of 100 μl was removed and boiled with loading buffer for sodium dodecyl sulfate-polyacrylamide gel electrophoresis (SDS-PAGE) analysis. The gel was visualized with Coomassie staining. The apparent bands were excised, partially digested with trypsin, and were analyzed by electrospray ionization (ESI)-ion trap mass spectrometry at Stanford University http://mass-spec.stanford.edu/.

### Stability assays of FliX and FlbD

Protein synthesis in cultures grown to mid-log phase was inhibited by addition of chloramphenicol to a final concentration of 3 mg/ml. One milliliter of cell culture was taken at 0, 15, 30, and 45 min after the addition of the antibiotic. Cell pellets were electrophoresed in 12% (w/v) polyacrylamide gels and were analyzed using anti-FlbD or anti-FliX antibodies.

### Site-directed mutagenesis of *fliX*

A fragment of 894 bp covering the coding sequence of *fliX *and its promoter region was amplified by PCR from *C. crescentus *chromosome and was inserted into pBBR1MCS to give raise to pZXfliX, which was then used as the template to create *fliX *mutants. For every mutant to be generated, two pairs of primers were designed to amplify the two fragments covering each side of the desired mutation site. The intended mutation sequence was overhung at the 5' end of the downstream fragment. For the convenience of manipulation, BamHI recognition sequence was engineered at the 5' end of the upstream fragment, and HindIII at the 3' end of the downstream fragment. The two fragments were then phosphorylated, treated with BamHI or HindIII, and inserted into pBBR1MCS to generate pZX series plasmids (Table 1). All mutants were confirmed by DNA sequencing.

### Protein expression analysis of FlbD and the FliX alleles

Overnight cultures of *C. crescentus *were transferred to fresh PYE media at a ratio of 1 to 10 (v/v) and were allowed to grow at 31°C until mid-log phase. Culture biomass was measured as optical density at 600 nm (OD_600_), normalized, and was subject to 14% (w/v) SDS-PAGE. After electrophoresis, protein profiles were transferred to nitrocellulose membranes and were detected using anti-FliX or anti-FlbD antibodies purified with affinity columns (AminoLink^® ^Plus Immobilization Kit, Thermo Fisher Scientific Inc., Rockford, IL, USA).

### Measurement of the transcription of flagellar genes

The pZX serial plasmids bearing various *fliX *mutants were introduced into the wild-type strain LS107 or the *ΔfliX *stain JG1172 via conjugation, along with the reporter genes *fliF-lacZ *or *fliK-lacZ*. β-Galactosidase activity was measured as described previously [40].

### Co-immunoprecipitation (co-IP)

Cells in middle log stage were harvested, normalized, and treated with 5 mg/ml lysozyme. The clear cell extract was incubated with Agarose-Protein A beads (Roche Applied Science, Indianapolis, IN, USA) to eliminate non-specific associated proteins. The pre-cleared cell lysate was then incubated overnight with Agarose-Protein A-anti-FlbD complexes prepared as instructed by the manufacturer. After extensive washing, the bead complexes were spun down, resuspended in SDS-PAGE sample buffer and were subjected to electrophoresis followed by immunoblotting with anti-FliX antibodies.

## Results

### FlbD forms stable *in vivo *complex with FliX

**Figure 1:**
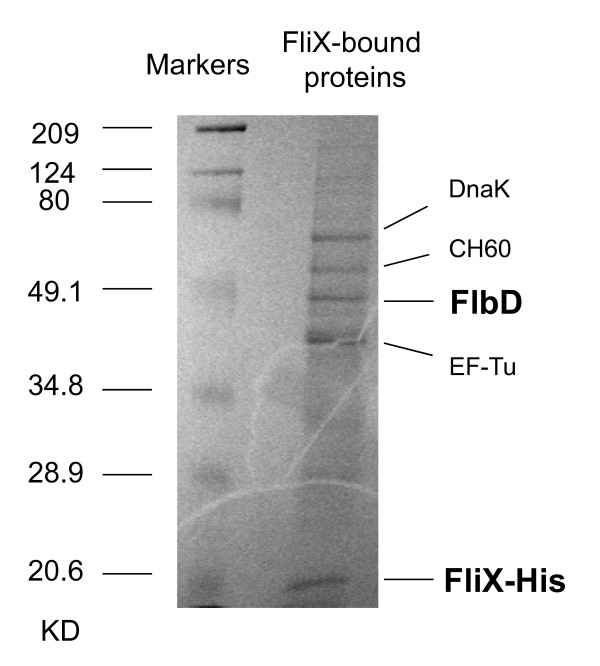
**Proteins bound to the sepharose beads coated with histidine-tagged FliX**. Purified FliX-His was conjugated to sepharose beads prior to incubation with cell lysis of LS107. The bead complexes were boiled with the sample buffer and were subject to SDS-PAGE analysis. The identities of the five major bands were determined by mass spectrometry.

Previous experiments have shown that FliX and FlbD interact in a two-hybrid assay [37], FliX can be precipitated from cell extracts of *Caulobater *by anti-FlbD antibodies, and that FliX regulates FlbD-activated transcription *in vitro *[35]. In order to gain further understanding of the physical recognition between the two and to find out whether there are other proteins associated with FliX-FlbD complex, we performed an affinity pull-down experiment in which cell extracts of *Caulobacter *were treated with sepharose beads coated with histidine-tagged wild-type FliX. Cellular proteins that physically associated to FliX were then retrieved from the bead complexes and resolved by electrophoresis (Figure 1). Five major bands with corresponding molecular weights of approximately 70, 60, 48, 44, and 19 kilodaltons were observed. Mass spectrometry analysis revealed that these proteins were heat shock proteins DnaK and CH60 (GroEL), FlbD, elongation factor EF-Tu, and FliX-His, respectively. Proteins DnaK, CH60, and EF-Tu are among the most abundant cellular proteins found in bacteria, including those possessing no flagellum. It is unlikely that these proteins would interact with FliX in a specific manner. Furthermore, when washing the sepharose bead complexes with phosphate buffer containing NaCl ranging from 0.3 to 2.65 M, these three proteins were readily released to the washing buffer throughout the salt gradient, whereas no FlbD or FliX protein could be washed off even with the highest salt strength used. The co-occurrence of FliX and FlbD in the sepharose bead complexes demonstrates that FlbD indeed directly interacts with FliX inside of *Caulobacter *cells, and that the affinity between the two proteins is remarkably high. We did not observe any other major specific component of the FlbD-FliX complex, although we cannot rule out the possibility that there might be transiently associated proteins, which are not detectable by the method described here.

### Interaction between FlbD and FliX is required for stabilizing each other *in vivo*

**Figure 2:**
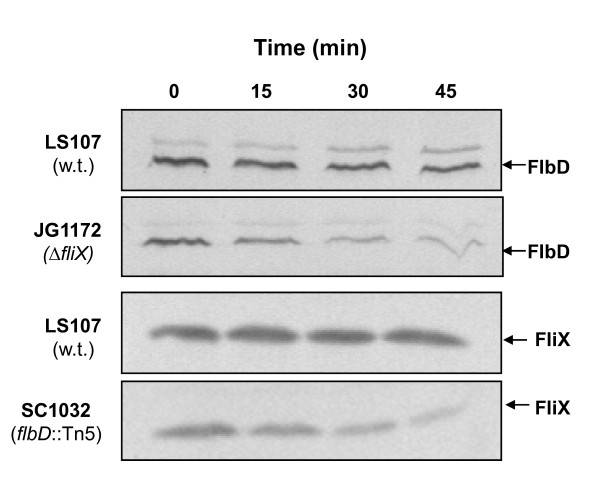
**Stability assays of FliX and FlbD**. Samples were periodically removed from cell cultures after the addition of chloramphenicol. Cell pellets were analyzed by SDS-PAGE followed by immunoblotting using anti-FlbD (upper panels) and anti-FliX (lower panels) antibodies.

The finding that FlbD and FliX form high affinity *in vivo *complexes motivated us to examine whether the two proteins depend on each other for existence. We assayed the half-life of each protein in a wild-type *Caulobacter *strain (LS107), a strain bearing a deletion in *fliX *(JG1172), and a strain having a Tn5 insertion in *flbD *(SC1032). Chloramphenicol was added to cell cultures at mid-log phase to inhibit protein synthesis, and the protein contents of FlbD and FliX were analyzed periodically. In strain LS107, both FlbD and FliX were stable; neither exhibited significant reduction in concentration following 45 min of exposure to chloramphenicol (Figure 2). In contrast, after 45 min, less than 40% of FlbD remained in strain JG1172. Likewise, a similar decrease in FliX level was evident in SC1032 cells. These results indicate that FlbD has a reduction in stability in the absence of FliX, and vice versa.

### Site-directed mutagenesis of FliX

**Figure 3:**
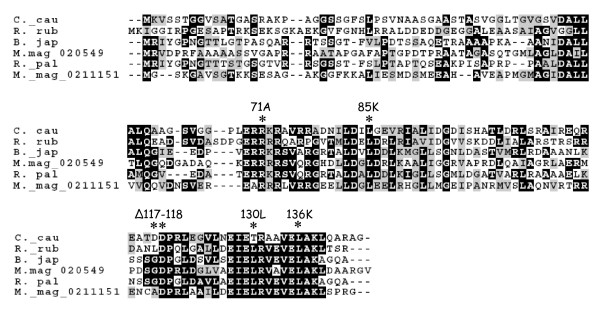
**Site-directed mutagenesis of *C. crescentus *FliX**. Homologs of *C. crescentus *FliX are aligned with CLUSTAL W 1.81 and are shaded with BOXSHADE 3.3.1. Black, identical residues; grey, similar residues; asterisks, sites of mutation. C._ cau: *C. crescentus*, R. rub: *Rhodospirillum rubrum*, B. jap: *Bradyrhizobium japonicum*, M. mag: *Magnetospirillum magnetotacticum*, and R. _pal: *Rhodopseudomonas palustris*.

To learn more about the interaction between FliX and FlbD, we performed site-directed mutagenesis with *fliX *and investigated the effects of mutations on FlbD activity. Both FlbD and FliX homologs are present in dozens of α-proteobacteria species that possess polar flagella. Among the FliX homologs, the central (residues 53-95) and carboxyl-terminal (residues 116-142) regions are highly conserved (Figure 3). To determine the roles of these regions in FliX functionality, five conserved sites were selected as the target sites for mutation: R71, L85, D117-D118, T130, and L136 (Figure 3). In the region from amino acids 69 to 73, there are five consecutive charged residues. This pattern is less common in protein sequences and may be important for FliX activity; so we chose to replace the central residue R71 with alanine to disrupt this pattern. We also deleted residues D117 and D118 in order to abolish these potential phosphorylation sites. In addition, we noticed that the 130th residue of FliX is a threonine, which is different from the majority of its homologs where a leucine is found. We then replaced T130 with an L and hoped to create a "super" FliX, because a conserved residue in a given position is often the most suitable one. Finally, we replaced L with K at sites 85 and 136 with the intention to disrupt any potential secondary structures of the conserved regions. Plasmid bearing either the wild-type or a mutant *fliX *allele, along with the *fliX *promoter region, was introduced into LS107 (wild-type strain) and JG1172 (*ΔfliX *strain) for further analyses.

### Role of conserved FliX residues in protein expression

**Figure 4:**
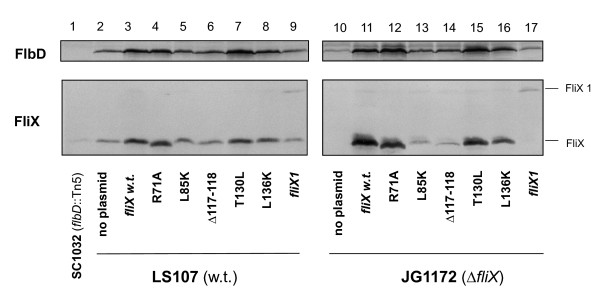
**Analysis of the cellular contents of the FliX mutants and FlbD**. Total proteins of LS107 and JG1172 cells expressing various *fliX *alleles were analyzed by SDS-PAGE prior to immunoblotting using anti-FlbD (upper panels) and anti-FliX (lower panels) antibodies.

We first examined the expression of the FliX alleles and FlbD. Cell extracts were subject to SDS-PAGE analysis followed by immunoblotting with anti-FliX and anti-FlbD antibodies (Figure 4). Strain SC1032 (*flbD*::Tn5) [41] and a constitutively active *fliX *allele (*fliX 1*), which carries an extended carboxyl terminus [38], were also included as controls. As was previously reported [36], the *flbD::*Tn5 cells possessed markedly reduced levels of FliX (lane 1); similarly, Δcells contained little FlbD (lane 10). These observations are also in support of the findings that FlbD and FliX interact with each other *in vivo *(Figure 1) and that the absence of either protein reduces the stability of the other (Figure 2). In both LS107 and JG1172 cells, FliX_R71A_, FliX_T130L_, and FliX_L136K _were present at levels comparable to wild-type FliX carried on a multi-copy plasmid (Figure 4, lanes 3 and 11). However, the concentrations of FliX_L85K _and FliX_Δ117-118 _in JG1172 cells were significantly reduced (greater than ten-fold) compared to other FliX mutants; the FlbD levels in these cells were also diminished (lane 13 and 14). Nevertheless, all mutants were successfully expressed in both wild-type and *ΔfliX *strains. We next examined the effects of these mutations on FlbD-mediated cellular processes.

### Role of conserved FliX residues in flagellar synthesis

**Figure 5:**
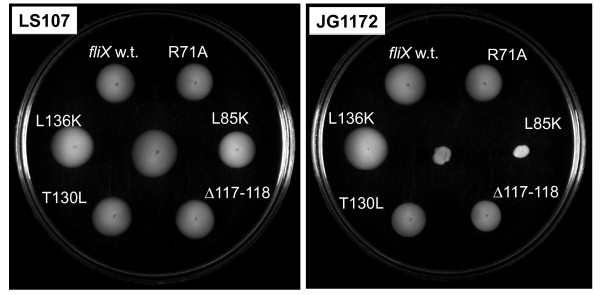
**Motility of the cells harboring various *fliX *alleles**. Cells were inoculated in motility agar and were incubated at 31°C for 3 days. Motile cells swarming away from the points of inoculation are visible as halos. Host strains containing no plasmid reside at the center of each plate.

Cells expressing each *fliX *allele were tested for motility using soft agar plates, on which motile cells swim away from the point of inoculation, forming a visible halo. In LS107 cells, the over-expression of either wild-type or mutant alleles of *fliX *from a multi-copy plasmid resulted in reduced swarm sizes, indicating that motility was slightly impaired by the over-expression (Figure 5). In JG1172 cells, all *fliX *alleles but *fliX*_L85K _were able to restore motility to the *ΔfliX *host (Figure 5); mutant *fliX*_Δ117-118 _resulted in the smallest swarm size. Since *fliX*_L85K _and *fliX*_Δ117-118 _were found at similar levels in JG1172 cells, it was intriguing to notice that the two mutants rendered distinctive physiological properties to their host cells.

**Figure 6:**
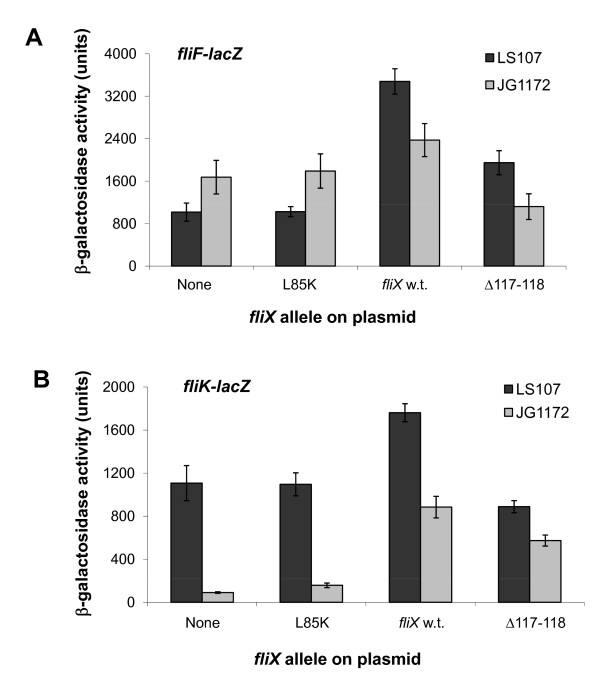
**Effects of *fliX *alleles on the transcription of flagellar genes**. Wild-type *fliX *and mutant alleles were introduced to LS107 or JG1172 cells containing reporter genes *fliF-lacZ *(A) or *fliK-lacZ *(B). Results of five independent experiments.

Previous experiments indicate that FliX functions as a positive regulator of FlbD activity [38]. In order to find out whether *fliX*_L85K _and *fliX*_Δ117-118 _can effectively regulate FlbD-mediated transcription of flagellar genes, the two mutants were introduced into LS107 and JG1172 cells that also contained either a *fliF*- (class II) or a *fliK-lacZ *(class III) transcriptional reporter fusion. When no *fliX *plasmid was involved, β-galactosidase activity generated from the *fliF *promoter was increased (Figure 6A) and from the *fliK *promoter (Figure 6B) was reduced in JG1172 cells compared to LS107 cells. This is in agreement with previous findings that FlbD represses the transcription of class II genes and activates the expression of class III genes [36]. In both LS107 and JG1172 backgrounds, transcriptional activity from either promoter in cells expressing *fliX_L85K _*was equivalent to that obtained in cells carrying no plasmid (Figure 6), suggesting that this *fliX *allele was completely impaired in activating FlbD. In both wild-type and *ΔfliX *cells, mutant FliX_Δ117-118 _regulated flagellar gene expression in a similar pattern as wild-type FliX did, albeit the overall activity of the reporter genes was lower, which could be due to the low cellular level of this mutant (Figure 4).

### Effect of conserved FliX residues on cell morphology

**Figure 7:**
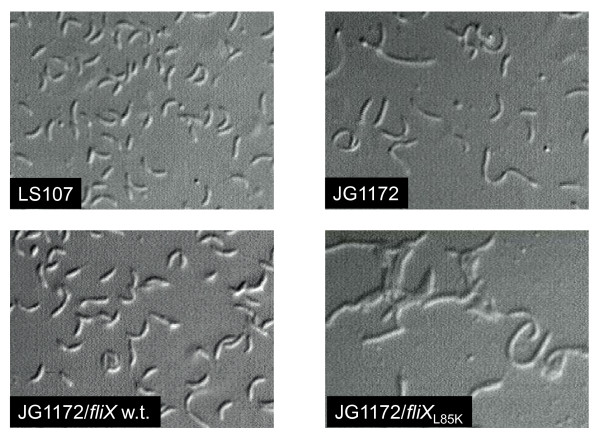
**Allele *fliX*_L85K _was unable to rescue the cell division defect of JG1172**. Cells harvested from overnight cultures were mounted on poly-L-lysine coated slides and examined by differential interference contrast (DIC) microscopy.

In *Caulobacter*, cells bearing mutations in early class II flagellar genes, including *fliX*, exhibit a cell division phenotype consisting of filamentous growth at the onset of late log phase [38]. As shown in Figure 7, the culture of JG1172 was dominated by filamentous cells, whereas JG1172 cells expressing the wild-type *fliX *gene had normal cell morphology. All *fliX *mutants, except *fliX_L85K _*(Figure 7), were able to restore the normal pattern of cell division in JG1172 cells. Once more, *fliX*_L85K _displayed no activity in complementing a physiological defect in *ΔfliX *cells.

### Role of conserved FliX residues in interaction with FlbD

**Figure 8:**
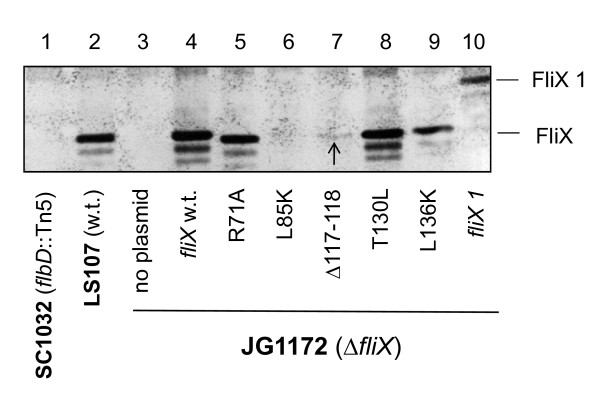
**Co-immunoprecipitation of FlbD and the FliX mutants**. Extracts of JG1172 cells expressing various *fliX *alleles were incubated with agarose beads coated with anti-FlbD antibodies. Proteins bound to the bead complexes were detected using anti-FliX antibodies following SDS-PAGE electrophoresis. The immunoblot was developed to an extended period of time to visualize the band of FliX_Δ117-118 _(indicated by the arrow).

Based on previous findings [35,37] and the new evidence of this study (Figure 1), it has been conclusively established that FliX and FlbD bind each other both *in vivo *and *in vitro*. The above genetic analyses revealed that although the cellular contents of FliX_Δ117-118 _and FliX_L85K _were similar in a *ΔfliX *background, they exerted distinctive effects on FlbD activity. Their ability to interact with FlbD must have played a role. To test this idea, we performed an immunoprecipitation experiment in which cell extracts of *Caulobacter *were treated with agarose beads coated with anti-FlbD antibodies, and elutes from the beads were probed with anti-FliX antibodies. As presented in Figure 8, mutants FliX_R71A_, FliX_T130L_, and FliX_L136K _interacted as well with FlbD as wild-type FliX did, if their cellular contents (Figure 4) were taken into consideration. In spite of the fact that FliX_L85K_, FliX_Δ117-118_, and FliX 1 were maintained at similar protein levels in JG1172 cells (Figure 4, lanes 13, 14, and 17), the precipitated amounts of these proteins were dramatically different (Figure 8, lanes 6, 7, and 10). Abundant FliX 1 and a small amount of FliX_Δ117-118 _were obtained, whereas no detectable level of FliX_L85K _was observed. This indicates that FliX 1 has a strong association to FlbD, FliX_Δ117-118 _binds to FlbD with a low affinity, and FliX_L85K _no longer interacts with FlbD. Since a large amount of FliX 1 was successfully precipitated, the results of this experiment reflect a lack of interaction between FlbD and FliX_L85K _rather than a lack of FliX_L85K _protein in the cell extracts.

## Discussion

The interaction between FliX and FlbD, two class II flagellar regulatory proteins in *Caulobacter*, represents a novel mechanism for regulating the activity of a σ^54 ^transcription factor. The activities of many such factors are regulated by the phosphorylation of a conserved aspartate residue in their receiver domains [42,43]. However, the receiver domain of FlbD diverges substantially from others [37]. For example, it lacks some key residues necessary for the phosphorylation process [44]. No corresponding cognate histidine kinase for FlbD has been identified so far, and FlbD is active in the absence of phosphorylation [30,34]. In addition, purified FliX can regulate FlbD-activated transcription *in vitro*, probably by affecting the oligomerization state of FlbD [35]. In this study, we further demonstrated that through a remarkably high affinity, the two proteins bind to each other to perform their regulatory activity and to escape the fate of premature degradation. Mutations in conserved regions of FliX could interrupt the recognition between the two and hence their activity.

The observed low concentrations of FliX_L85K_, FliX_Δ117-118_, and FliX 1 in JG1172 cells may be caused by their intrinsically low expression levels or their short half-life, or a combination of both. DNA or mRNA sequences of the alleles may carry intrinsic defects that inhibit the transcription or translation efficiency of the mutated genes. It is also possible that the mutations unfortunately expose target sites to intracellular proteases, making the gene products prone to degradation. Lack of protection from FlbD may also play a role in the case of FliX_L85K_. No matter what might be the main cause, the final result is that the cellular levels of the three are about the same (Figure 4). Nevertheless, their differential binding affinities to FlbD lead to dramatically different physiological outcomes. FliX_L85K _completely losts the ability to interact with FlbD and exerts no influence to FlbD-mediated cellular processes. The fair amount of cellular FliX_L85K _(Figure 4) does not benefit the *ΔfliX *host in any observable way (Figure 5, 6 and 7). The mutation must have altered the gross structure of FliX and thus prevented an effective binding to FlbD. FliX_Δ117-118 _can still interact with FlbD to a certain degree; therefore, it is largely functional in regulating FlbD activity (Figure 5 and 6). With a strong affinity to FlbD, FliX 1 becomes constitutively active; it turns on the transcription of class III/IV genes in the absence of the class II basal body [37,38]. The other three mutations, R71A, T130L, and L136K cause no significant effect to the expression of FliX, the interaction with FlbD, and hence the regulatory activity of the two partners.

Since the three dimensional structure of FliX (or a homolog) remains to be solved, it is still unclear which residues or regions of FliX and FlbD are in direct contact. An alanine scanning analysis should be helpful to probe the structural basis of the interaction. Relevant insights could also be gathered by selection for extragenic suppressors in FlbD that are specific to loss-of-function FliX alleles like FliX_L85K_.

## Conclusions

Direct association of FliX and FlbD is required for their regulation on flagellar synthesis and other developmental events in *Caulobacter*. FliX and FlbD form high affinity complexes under physiological conditions, which is essential for the *in vivo *stability of each protein. Highly conserved regions of FliX are critical for binding to FlbD. Mutations in these regions could severely impact the recognition between the two and compromise their regulatory activity.

## Authors' contributions

JWG conceived and coordinated the study and helped to draft the manuscript. RJD performed the protein stability assay. ZX carried out the rest experiments and drafted the manuscript. All authors participated in experiments designs and data analyses. All authors read and approved the final manuscript.
